# Fasciculoventricular pathways—A rare and innocent variant: A Retrospective study focusing on clinical and electrophysiologic characteristics

**DOI:** 10.1111/anec.12913

**Published:** 2022-01-02

**Authors:** Suat Gormel, Salim Yasar

**Affiliations:** ^1^ Department of Cardiology Gulhane Research and Training Hospital Ankara Turkey

**Keywords:** accessory pathway, fasciculoventricular, supraventricular tachycardia

## Abstract

**Background:**

Fasciculoventricular pathways (FVPs) are variants of pre‐excitation syndrome which were investigated insufficiently because of its rarity.

**Objective:**

This report aimed to represent one of the largest series of FVP, focusing on its clinical and electrophysiological properties.

**Methods:**

We analyzed retrospectively 26 consecutive patients who underwent electrophysiological study (EPS) for FVP between January 1998 and June 2020.

**Results:**

Among 1437 patients with accessory pathways, 26 had FVP (1.80%). All the 26 patients (100%) were males, with a mean age of 22.15 ± 3.50 years (range, 20–34 years). In the baseline electrocardiograms of the patients with FVP, pre‐excitation and transitional zone were seen in leads V_2_–V_4_. During EPS procedures, normal AH interval and shortened HV interval were detected. All the patients had AH prolongation after atrial pacing due to atrioventricular (AV) nodal delay without change in pre‐excitation degree. Five of the FVP patients (19.2%) had extra accessory pathways, all of which were ablated successfully while the FVPs were followed clinically.

**Conclusion:**

Fasciculoventricular pathways are uncommon variants of pre‐excitation syndrome; therefore, they should be diagnosed correctly and followed up noninvasively to avoid damages.

## 1 INTRODUCTION

Fasciculoventricular pathways (FVPs) are known to be rare variants of pre‐excitation syndrome. When we focused on the literature, based on electrophysiologic studies in patients presenting with some symptoms such as palpitation, it was reported 1.2% to 5.1% of pre‐excitation syndrome (Josephson, 2002; Sternick et al., 2003), but some studies, for example, a survey in Japanese students, showed FVP more common than Wolff–Parkinson–White Syndrome (WPW). Similarly in an anatomic study, FVP was found in 1 of 20 heart specimens, suggesting FVP might be much more common than anticipated. The prevalence of FVP among asymptomatic patients has not been reported, and in context of these data, it can be said that it is an overlooked arrhythmia.

They are accessory connections taking off from the bundle of His or the fascicles and inserting into the ventricles. FVPs do not give rise to reciprocating tachycardias being bystander bundles (Anderson et al., 2020; Gallagher et al., 1981; Sternick & Wellens, 2006). Electrocardiogram (ECG) characteristics of an FVP are consisted of a minimal pre‐excitation pattern with a normal QRS frontal plane axis and a variable PR interval (Anderson et al., 2020; Gallagher et al., 1981; Sternick & Wellens, 2006). It is crucial to correctly identify FVP and distinguish them from anteroseptal bypass tracts. FVP and anteroseptal accessory pathway (AP) can mimic each other, and during catheter ablation of a misdiagnosed anteroseptal AP, atrioventricular (AV) nodal conduction may be disrupted inadvertently (Anderson et al., 2020; Gallagher et al., 1981; Sternick & Wellens, 2006). This study aimed to describe long‐term FVP in a cohort of patients, focusing on its clinic and electrophysiologic properties.

## 2 MATERIALS AND METHOD

Our study is a retrospective analysis, and our population consisted of 26 consecutive patients who underwent electrophysiological study (EPS) and were diagnosed with FVP between January 1998 and June 2020 in Gulhane Faculty of Medicine, Department of Cardiology, Ankara, Turkey. The patients’ epidemiological characteristics, clinical features, medical treatments, ECGs, echocardiographic evaluations, and baseline ECG/EP characteristic data were analyzed. There was no exclusion criterion. Local ethics committee approved the study.

### 2.1 ECG interpretation

The diagnosis of FVP was made using the following ECG criteria (i) a shorter QRS duration (<120 ms); (ii) a not so short PR interval (110–120 ms); (iii) a flat or negative delta wave in V_1_; (iv) a narrow delta/R wave in V_2_; and (v) S‐wave amplitude <20 mm in V_1_.

### 2.2 EPS procedure

Written informed consent was obtained from all individual participants included in the study. The study was approved by the Institutional Ethical Committee. All antiarrhythmic drugs were discontinued 4–5 half‐lives before ablation. Ablation procedures were performed under local anesthesia in a fasting state. Three catheters were introduced through the right femoral vein and were positioned at the high right atrium (HRA), coronary sinus, and His region. The catheter at His position or catheter at HRA was shifted to the right ventricle when necessary. Intracardiac signals were filtered at 20–500 Hz, and amplification gains were 10–80 mm/mV. All signals were displayed and acquired on an electrophysiological recording system (EP TRACER 2^®^ system, CardioTek BV). Baseline AH, HV, and BCL intervals were measured; then, programmed atrial and ventricular electrical stimulation was performed.

Electrophysiological diagnosis of FVP was made using the following criteria:

1. During incremental atrial pacing and atrial extrastimulus testing, prolongation of AH interval was observed whereas HV interval remained unchanged.
2. Retrograde conduction was concentric and decremental.
3. No tachycardia was induced.

### 2.3 Statistical analysis

This was a descriptive study in which the categorical variables were represented as absolute numbers and percentages. Continuous variables were represented as mean ± standard deviation. Statistical analyses were performed using SPSS software for Windows (version 20.0; SPSS, Inc).

## 3 RESULTS

We evaluated 8233 patients who underwent EPS. Among the cases, we established 1437 patients with 1497 APs. Among the 1437 patients, 26 had FVP (1.80%). All the 26 patients were males (100%). The mean age was 22.15 ± 3.5 years (range, 20–34). The patients were clinically asymptomatic except the ones with accompanying arrhythmia. All the patients had a resting 12‐lead ECG revealing minimal pre‐excitation during sinus rhythm. None of the patients had structural abnormalities of the heart noted in transthoracic echocardiography.

**FIGURE 1:**
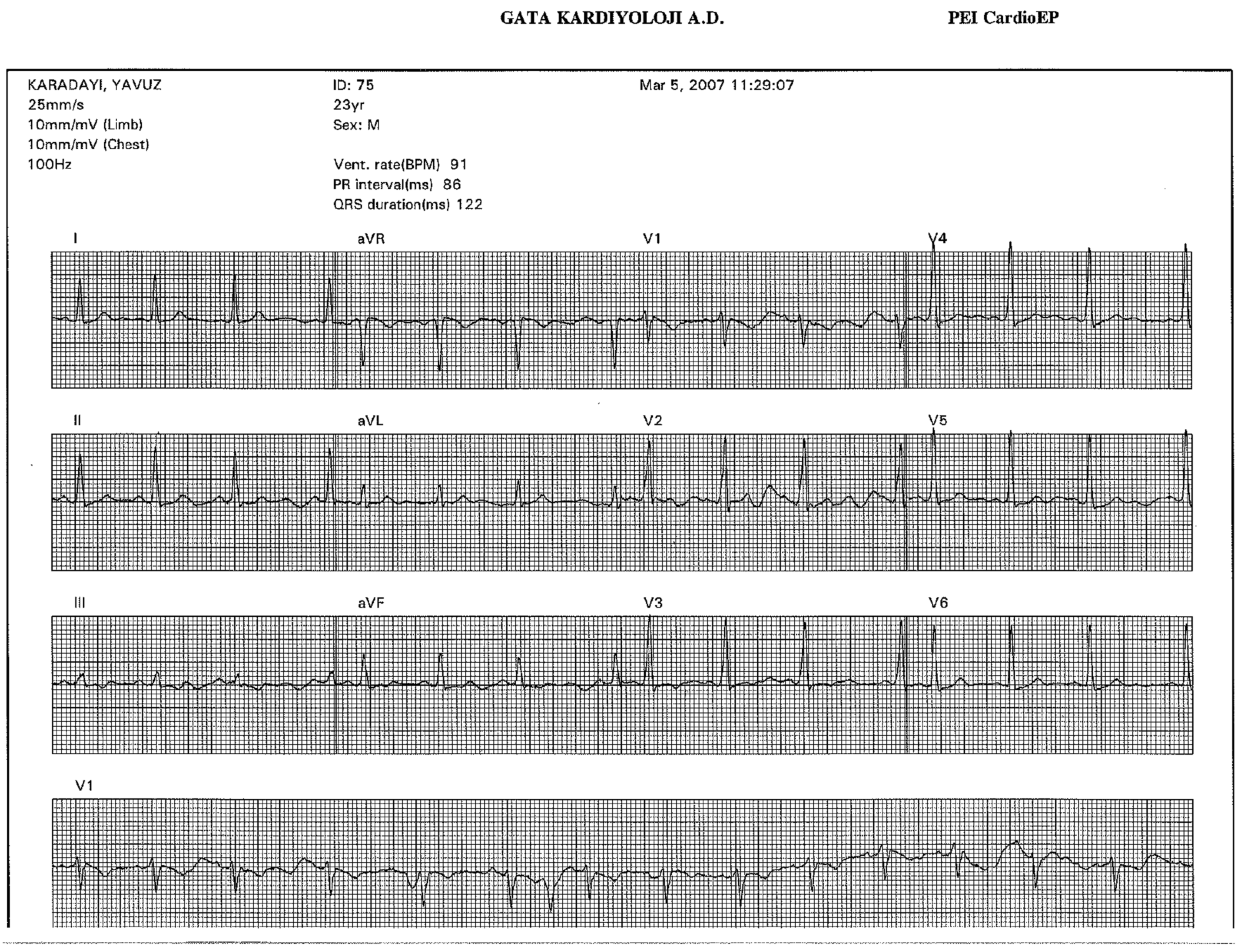
Patient's baseline electrogram. Sinus rhythm at 91 beats per minute. PR interval of 110 ms, QRS interval of 118 ms, and QTC interval of 456 ms. Evidence of pre‐excitation demonstrated by slurred QRS upstroke (delta wave) in leads V_3_‐V_6_

The electrocardiographic findings of FVP patients were as follows: Mean PR interval was 112.46 ± 10.17 ms (range 90–130 ms), mean QRS complex width was 95.80 ± 15.15 ms (range 80–118 ms), QRS transition (R/S>1) occurred between V_2_ and V_4_ precordial leads, the polarity of delta waves were flat in 65.39% of patients and negative in 34.61% of patients in V_1_ lead, mean amplitude of S wave was 9.35 ± 2.59 ms (range 5–13 msn), and 42.3% of patients had notching in the descending limb of S waves in V_1_ lead (Figure 1).

**FIGURE 2:**
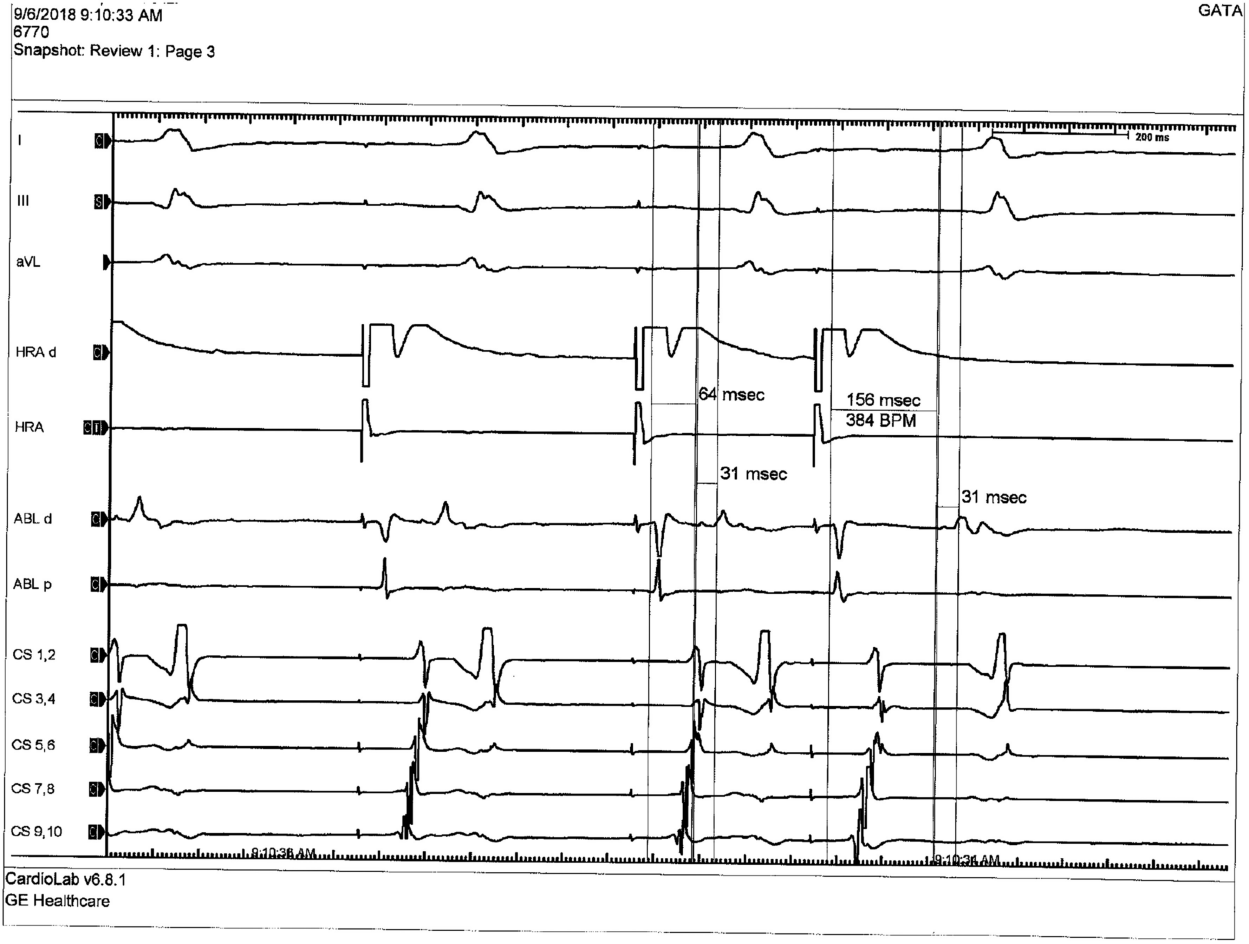
Intracardiac recordings during pacing from the para‐Hisian region. During incremental atrial pacing, prolongation of the AH interval and constant degree of pre‐excitation with fixed HV interval was observed

During EPS procedures in patients with FVP, pre‐excitation with normal PR and AH intervals and short HV intervals was shown. All patients had AH prolongation after atrial pacing due to AV nodal delay without change in pre‐excitation degree (Figure 2). Ventricular pacing was performed from right ventricular apex, and concentric V‐A conduction was established in all study patients. Programmed electrical stimulation from the atria and ventricle did not reveal any inducible tachycardias in any of the patients related to FVP, and no ablation was performed.

Of the 26 patients, 4 had AV nodal reentrant tachycardia (AVNRT), 1 had AV reentrant tachycardia (AVRT), and 1 had both AVRT and AVNRT. The two patients with AVRT had both left lateral localized APs. Coinciding arrhythmia was ablated successfully in the same session with RF ablation in five patients, except in one patient whose procedure was postponed because of the patients’ choice.

No complications were encountered during the EPS procedure. All the patients were discharged in the following 24 hours and followed up clinically. From the national database of mortality, we reached the mortality data of the patients and revealed that no deaths were occurred related to an arrhythmia through twenty years. Baseline characteristics, ECG features, and electrophysiological data of the patients are shown in Table 1.

**TABLE 1:** Baseline characteristics, ECG features, and electrophysiological data

Sex (males) (*n*, %)	26/26 (100)
Age, mean (SD)	25.65 ± 10.56 (17–66)
Structural heart abnormalities (*n*, %)	0/26 (0)
Other associated tachycardias	
AVNRT (*n*, %)	4/26 (15.38)
AVRT (*n*, %)	1/26 (3.84)
AVRT and AVNRT (*n*, %)	1/26 (3.84)
Baseline ECG features	
Manifest pre‐excitation during sinus rhythm (*n*, %)	26/26 (100)
PR width (ms), mean (SD)	112.46 ± 10.17 (90–130)
QRS width (ms), mean (SD)	95.80 ± 15.15 (80–118)
Transition zone	V_2_‐V_4_
Flat delta wave in V_1_ (*n*, %)	9/26 (34.61)
Negative delta wave in V_1_ (*n*, %)	17/26 (65.39)
S‐wave amplitude in V_1_, mean (SD)	9.35 ± 2.59 (5–13)
Notching in the descending limb of the S wave in V_1_ (*n*, %)	11/26 (42.30)
Electrophysiological data	
Procedure time (min), mean (SD)	65.81 ± 22.13 (43–130)
Basic cycle length (ms), mean (SD)	733.96 ± 118.78 (480–1000)
AH interval (ms), mean (SD)	73.04 ± 18.75 (43–111)
HV interval (ms), mean (SD)	27.76 ± 7.04 (12–35)
Wenckebach point (ms), mean (SD)	334.55 ± 48.57 (270–450)

Abbreviations: AVNRT, AV nodal reentrant tachycardia; AVRT, atrioventricular reentrant tachycardia.

## 4 DISCUSSION

The septal region of the heart has anatomic complexity owing to the fact that it includes 30% of all accessory AV pathways as well as FVP. In a diverse way, FVPs are innocent variants and never involved in reciprocating tachycardia circuits such as other accessory AV pathways. Even though in asymptomatic patients catheter ablation of the APs successfully eliminates the low risk of sudden death associated with the condition, FVPs are exceptionable and its ablation is generally not beneficial for risk reduction.

More recently, limited data originating from some case reports suggested that FVP may also be involved in reentrant circuits. In contrast to the well‐reported FVP, these patients do not show ventricular pre‐excitation in a sinus rhythm ECG and the tachycardia has AV dissociation and short HV interval, apart from an eccentric AV conduction during tachycardia (Chung et al., 2019; Higuchi et al., 2021; Tritto et al., 2020).

The current consensus recommends that upon discovery of ventricular pre‐excitation, first‐line noninvasive diagnostic methods such as exercise stress test should be done to assess the persistence of pre‐excitation to evaluate the mortality risk. Our hospital’s EPS archives by a large majority, as once being a military hospital, consisted data of asymptomatic male military staff who had been referred for medical evaluation of pre‐excitation. After risk stratification with noninvasive tests, the high‐risk group with unsatisfied results were evaluated using invasive methods as recommended (Pediatric and Congenital Electrophysiology Society (PACES) et al., 2012; Brugada et al., 2020) Management of asymptomatic athletes or high‐risk professionals such as military staff with a WPW ECG pattern remains a dilemma. Identification and excluding asymptomatic AP‐like FVP is the major concern.

Our results of ECG analysis associated with lead V_1_ provide the following useful findings in the diagnosis of FVP consistent to the literature: not so short PR interval, S‐wave amplitude <20 mm, and flat or negative delta wave. Although notching in the descending limb of S wave was noticed as other valuable electrocardiographic findings in the diagnosis of FVP, current literature supporting these data is limited (Oh et al., 2005). These findings can be advantageous in differential diagnosis in advance of invasive diagnostic procedures in context of avoiding unnecessary and potentially harmful catheter manipulations or ablations.

Although electrocardiographic findings are helpful, the gold standard for diagnosis of FVP is EPS. Several authors have suggested that FVP can be safely diagnosed by baseline ECG, with or without additional pharmacologic testing. However, the only independent predictor for diagnosis of the FVP they had found was a larger amplitude of the delta wave (Oh et al., 2005; O'Leary et al., 2019; Sternick et al., 2003; Suzuki et al., 2014).

Our study group is one of the largest series for FVP retrieved over a 20‐year period. Our incidence of FVP in patients with pre‐excitation was 1.8%, similar to that of the literature ranging from 1.2% to 5.1% (Josephson, 2002; Sternick et al., 2003). In fact, in our hospital, patients other than military staff were also examined. However, the asymptomatic patients with pre‐excitation who needed further evaluation as high‐risk group were generally military personnel.

Five of our FVP patients with FVP (19.2%) had extra APs which was lower than the previous reports (Ganz et al., 1998; Kottkamp et al., 1996; Sallee & Van Hare, 1999; Sternick et al., 2003; Sternick & Wellens, 2006). Considering all of these previous reports with FVP, the incidence of associated AVNRT is 33% (Sternick et al., 2003). The reason of our lower incidence for AVNRT is probably due to our effort for evaluating asymptomatic high‐risk profile patients whose FVPs were mostly diagnosed incidentally.

Multiple APs are estimated to occur in up to 13% of WPW patients (Colavita et al., 1987). Thus, when evaluating a pre‐excitation especially an anteroseptal one, FVP should be kept in mind. As an uncommon variant, FVPs are not involved in reciprocating tachycardia circuits and should only be managed clinically. However, FVP may cause electrocardiographic curiosities while associating with other additional rapidly conducting AV bypass tracts, and they may be misdiagnosed as para‐Hisian APs. Consequently, misinterpretation may result in undesirable conditions (Suzuki et al., 2014). FVP ECGs may be misinterpreted as WPW with anteroseptal APs. They have pre‐excitation and transitional zone in leads V_2_–V_3_ due to their anteroseptal location just like WPW patients. Therefore, it is generally compelling to distinguish FVP patients based on ECG evidence (Alencar Neto et al., 2019). In a study by Sternick et al. comparing the ECG findings of FVP and WPW with anteroseptal AP, QRS transition in the precordial leads occurred earlier mainly in lead V_2_, and QRS width and PR interval were shorter in patients with FVP. In the present study, early QRS transition in the precordial leads did not observed. On the other hand, a shorter QRS width and shorter PR interval were determined as differentiating features for FVP patients from septal bypass tracts (Sternick et al., 2005). FVPs are associated with various genetic syndromes; however, a genetic disorder caused by PRKAG2 gene mutation is the special group which should be considered every time because of its specific and noteworthy clinical manifestations. This specific group is inherited genetically and associated with glycogen storage cardiomyopathy such as the AP disease familial WPW syndrome. Patients with FVP and the PRKAG2 mutation may present with typical clinical, electrocardiographic, and electrophysiological features. These patients, who have a more malignant prognosis, experience a high incidence of left ventricular hypertrophy, sinus bradycardia, complete AV block, and atrial arrhythmias. Although FVP patients without structural heart disease have a rather good prognosis, patients with PRKAG2 mutation have a high incidence of conduction disturbances requiring pacemaker implantation (Ali et al., 2015; Hoffmayer et al., 2020; Sternick et al., 2011).

Because of once being a military hospital, our study group mostly consisted of healthy military staff who were examined before in recruiting examinations for possible major heart diseases. Possible FVP patients with LV hypertrophy, conduction disturbances, or atrial arrhythmias were excluded in the examinations. Therefore, we did not screen for PRKAG2 gene mutations in our hospital because we considered our FVP patients to be low risk. Furthermore, we did not encounter any fatal clinical manifestation in the following time. Thus, according to our clinical experience, we suggest that PRKAG2 gene mutation screening be kept for FVP patients with high‐risk features.

## 5 LIMITATIONS

This study is a single‐center retrospective study in which the epidemiological characteristics of the patients and findings of electrophysiological procedures were retrieved from institutional archive. Majority of our study group included male military staff referred for medical evaluation, inconsistent with the general population.

## 6 CONCLUSION

Fasciculoventricular pathways are uncommon variants of pre‐excitation syndrome which should be considered as a definitive diagnosis and followed up noninvasively when it is diagnosed to avoid damages. In this setting, EPS is gold standard procedure facilitating the diagnosis of FVP which allows for adequate risk stratification. FVP patients with PRKAG2 gene mutations should be evaluated for high‐risk clinical outcomes.

## CONFLICT OF INTEREST

The authors declare that they have no conflict of interest.

## AUTHOR CONTRIBUTIONS

Conception and design of the research: Suat Gormel, Salim Yasar. Data collection and writing the initial manuscript: Suat Gormel, Salim Yasar. Critical revision of the manuscript for intellectual content: Suat Gormel, Salim Yasar. All contributions critically revised the final manuscript.

## ETHICAL APPROVAL

This study was conducted in accordance with the Declaration of Helsinki and approved by the ethics committee of Gulhane Research and Training Hospital.

## Data Availability

Data available on request due to privacy/ethical restrictions.
